# Reliability of a new loaded rolling wheel system for measuring spinal stiffness in asymptomatic participants

**DOI:** 10.1186/s12891-019-2543-y

**Published:** 2019-04-24

**Authors:** Maliheh Hadizadeh, Greg N. Kawchuk, Eric Parent

**Affiliations:** 1 Department of Physical Therapy, Faculty of Rehabilitation Medicine, University of Alberta, 3-48 Corbett Hall, Edmonton, AB T6G 2G4 Canada; 2 Sports Science and Clinical Biomechanics, University of Southern Denmark, Campusvej 55 5230, Odense, M Denmark; 3 Department of Physical Therapy, Faculty of Rehabilitation Medicine, University of Alberta, 2-50 Corbett Hall, Edmonton, AB T6G 2G4 Canada; 4 Department of Physical Therapy, Faculty of Rehabilitation Medicine, University of Alberta, 3-44 Corbett Hall, Edmonton, AB T6G 2G4 Canada

**Keywords:** Reliability, Test-retest, Spine, Stiffness, VerteTrack

## Abstract

**Background:**

Few, if any, patient reported symptoms have been shown to be related to objective measures of spine function. Recently, patient-reported measures of disability following spinal manipulative therapy have been associated with an immediate decrease in spinal stiffness obtained by instrumented L3 indentation. Given this novel relation, we anticipate that stiffness measures obtained from locations in addition to L3 may yield valuable information. As such, our research team has developed a new technique to acquire stiffness data continuously over an entire spinal region. The reliability of stiffness measurements obtained by this new technique has yet to be quantified.

**Methods:**

Continuous stiffness testing employs a weighted roller that moves uninterrupted over the spine while measuring the resulting spinal deflection along a subject-specific, laser-defined trajectory. A volunteer sample of asymptomatic participants were assessed in 2 sessions occurring 1 to 4 days apart, with each session scheduled at the same time of day. Each session consisted of 3 trials each beginning at a baseline of ~ 17 N then progressing to a maximally tolerable load as defined from pre-test familiarization trials (~ 61, 72 or 83 N). Reliability was evaluated with the intraclass correlation coefficient, the standard error of measurement and Bland & Altman analysis.

**Results:**

A total of 17 asymptomatic participants (mean age 29.2 +/− 6 years, 53% female) took part in the study. Overall, the within and between-session reliability of lumbar spine stiffness measures at the maximal tolerable load was excellent ranging from 0.95–1.00 and good to excellent ranging from 0.82–0.93, respectively. Trial averaging was found to reduce standard error of measurement by a mean of 35.2% over all measurement conditions compared to a single trial. Bland and Altman plots for agreement in lumbar spine stiffness measurements varied from − 0.3 +/− 1.2 at unloaded condition to − 0.2 +/− 1.2 at loaded condition. Data from two participants were removed due to the development of back pain between two sessions.

**Conclusion:**

This study introduced a new technique for measuring spinal stiffness over an entire spinal region in asymptomatic human participants. The new technique produced reliable measurements quantifying the load-displacement values for within-session and between-session assessments.

## Background

A significant decrease in the mobility of lumbar spine has been reported as a common sign in individuals with low back pain (LBP) [1]. Previous studies showed that there is a relation between pain and spinal stiffness [2]. With this in mind, spinal stiffness assessment has become a common practice in clinical settings in the management of patients with spine-related pain [2, 3]. Practitioners routinely evaluate spinal stiffness to provide a basis for diagnosis, prognosis and treatment decision-making [2] as well as to monitor the efficacy of treatments such as manipulation [4]. Typically, the clinical assessment of spinal stiffness involves a manual test where a clinician applies pressure in a posteroanterior (PA) direction to the spinous process of interest [5]. As stiffness magnitude cannot be quantified precisely with this manual technique, a categorical rating system is often used where the segment of interest is classified as hypomobile, normal, or hypermobile, based on the clinician’s perception of stiffness [5]. Unfortunately, prior studies have shown that clinical judgment of PA testing is highly variable in terms of the magnitude [6], direction [7] and the speed of applied load [2] as well as the discrimination threshold for stiffness perception [8].

Due to low levels of reliability and high variability related to clinical evaluation of spine stiffness, mechanical tools have been developed to quantify the applied loads and tissue displacement that occur during PA testing [2, 3, 5] the majority of which assess force-displacement at a static location. Using this approach, we have shown previously that patient-reported measures of disability following spinal manipulative therapy (SMT) are associated with an immediate decrease in spinal stiffness obtained by instrumented L3 indentation (R = 0.3) [9, 10]. Given this novel relation, we anticipate that stiffness measures obtained from locations in addition to L3 may yield valuable clinical information. We also hope insights into this area may lead to better management of symptoms of LBP.

As such, our research team has developed a novel device to improve on single-site spinal indentation by employing a loaded rolling wheel system. The reliability of stiffness measurements obtained by this new technique has yet to be quantified. Therefore, the objective of this study was to determine the within- and between-session reliability of lumbar stiffness measurements in asymptomatic participants using this new loaded rolling wheel system (VerteTrack™, VibeDx Corporation, Canada).

## Methods

### Participants

A total of 17 consecutive volunteers were recruited using flyers distributed on campus at University of Alberta. The sample size calculation was based on an estimate used specifically for reliability studies [11]. Thirteen subjects are needed to detect an ICC of 0.9 with three replications (k = 3) against a Null-hypothesis of 0.7.

Study participants included asymptomatic males and females between the ages of 18 and 60 with no history of thoracic and lumbar pain within the last 6 months. Participants were excluded from the study if they could not tolerate the stiffness testing procedure, lay prone for 20 min, or had a history of the following: scoliosis, congenital spinal disorders, prior thoracic or lumbar surgery, spondylolisthesis, cauda equina syndrome, current pregnancy, severe respiratory disease, severe trauma, or a medical ‘red flag’ such as cancer, spinal infection, fracture, or systemic disease.

### Examiner

A research assistant with 6 years of clinical experience in physical therapy and 1 year of experience using the testing device collected all measurements.

### Continuous stiffness testing device

**Fig. 1:**
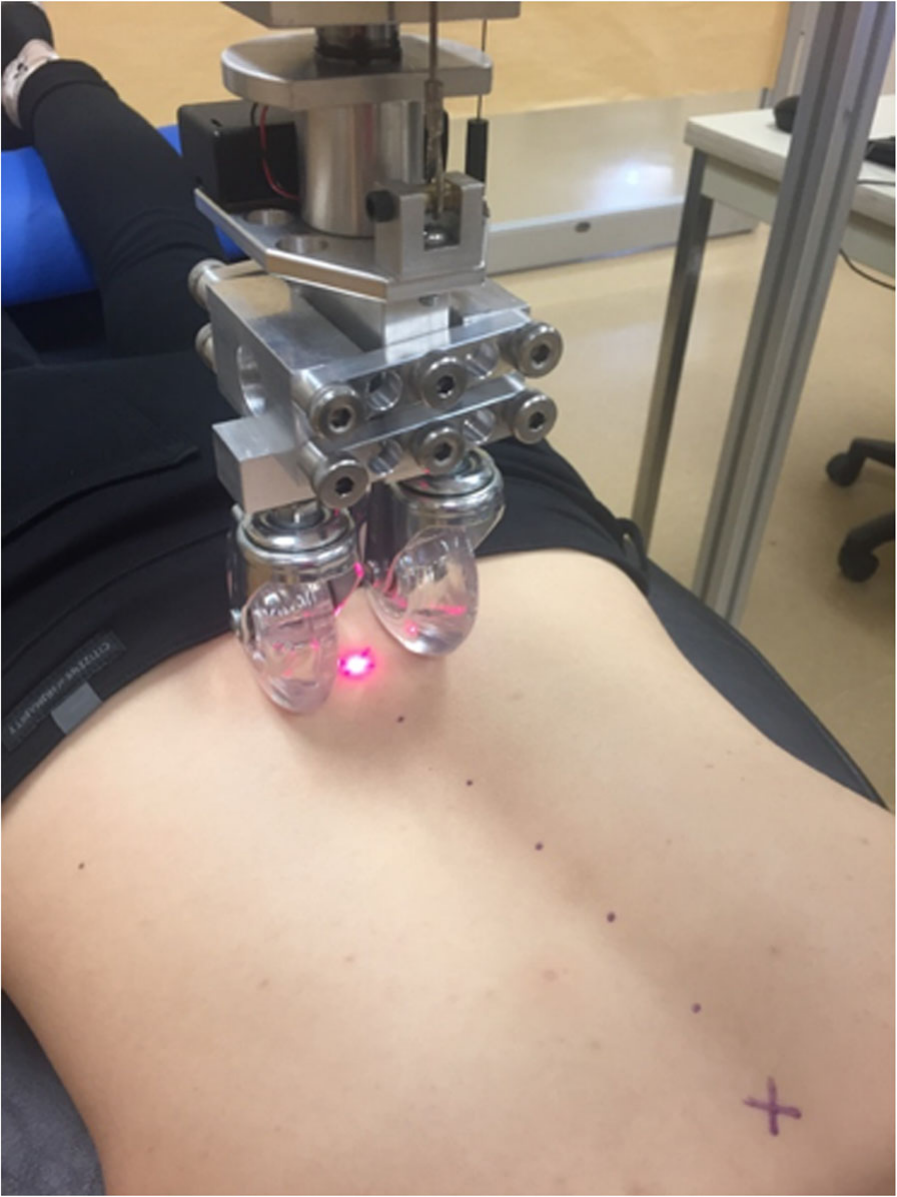
Superior view of the device showing the laser/wheel assembly

The lumbar PA trunk stiffness was assessed with a mechanical device (Fig. 1) whose comfort and safety has been studied in a sample of young adults previously [12]. The device consists of a solid, cube-shaped aluminium frame that provides a rigid support for the roller apparatus. The roller apparatus consists of a vertical rod suspended within a linear bearing to permit near-frictionless vertical translation of two rolling wheels of 70 mm diameter with variable inter-wheel spacing (typical 29 mm, ranging from 16 to 54 mm). This inter-wheel spacing adjustments allows the wheels roll over the most prominent part of the paravertebral tissues and not over the spinous processes. Inter-wheel spacing was obtained for each participant by measuring the distance between the apex of the paraspinal tissues using a ruler.

**Fig. 2:**
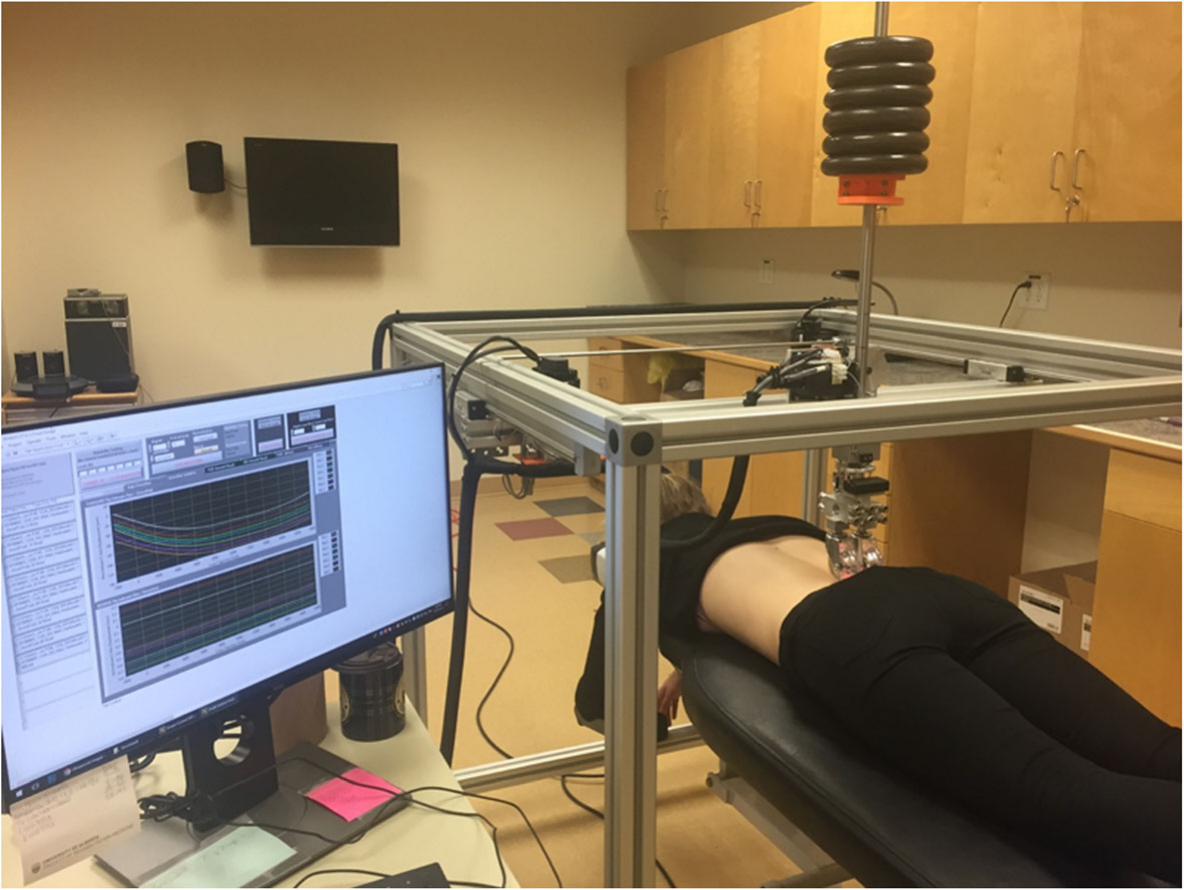
Continuous stiffness testing device with participant positioned for the measurement of lumbar spine stiffness. The device measures displacement which is produced by loads applied to the vertical rod. The software quantifies stiffness values as a ratio between the applied force and the resultant displacement. The weight of the unloaded roller is ~17 N. Each additional mass increment is ~11 N

A stepping motor system (resolution = 0.007 mm) (National Instruments, USA) is used to position the roller along the X (longitudinal, cephalad/ caudal), Y (transverse, left-right) axes with built-in encoders to confirm motor position. The vertical Z axis employs a stepper motor system (Stepperonline.com, China) that is connected to a cable which raises and lowers the rollers in conjunction with a string potentiometer to quantify vertical position (resolution = 0.020 mm, TE Connectivity, USA). Control of all motors and acquisition of signals is provided by in-house coding using LabVIEW (National Instruments, USA, Fig. 2). Using this controlling software, it is possible to position the roller in three dimensions. This allows clinicians to manually position the rollers to specific positions along the spine and use a laser pointer mounted on the vertical rod. The laser pointer allows alignment of the rollers to each of the spinous processes of the targeted segments while the device stores the resulting X and Y coordinates. The device then stitches these coordinates together to create a XY trajectory for the wheels to follow. The system then lowers the roller onto the participant and adds additional slack to the Z-axis cable. The roller is then free to move vertically in response to the tissue resistance found along the predefined X-Y trajectory. By repeating this process with additional mass attached to the roller, a continuous measure of the PA bulk deformation of any spinal region, and hence stiffness, can be quantified.

### Study procedures

Each participant was assessed in 2 separate sessions occurring 1 to 4 days apart. Both sessions were conducted at the same time of day. Prior to testing, consenting participants completed self-reported questionnaires on demographics and medical history as well as an 11-point numeric pain rating scales (NPRS-11) before and after each session.

Standardized instructions were given to the participants before testing which included information about how to hold their breath during testing (held expiration), to remain still during testing and to provide feedback if they experienced pain or felt they were resisting the roller wheels. An inter-wheel spacing of 29 mm was used for all participants in both sessions.

To begin using the device, the examiner first manually identified and marked each spinous processes from S1 to T12. The examiner then used the laser system described previously to generate an XY trajectory for the wheels to follow (Fig. 1). During subsequent stiffness testing, participants were instructed to hold their breath at the end of a normal exhalation for approximately 10s while the device was lowered on to the first trajectory point (S1) and the roller was then automatically moved through the remaining XY trajectory points with the roller free to move vertically in response to spinal topography and tissue resistance. Approximately 10s later, at the last trajectory point (T12), the device was automatically lifted off and returned to the first trajectory point just above S1 while the participant was instructed to continue breathing normally. This process was then repeated with increasing mass attached to the roller with testing ending at either the addition of ~ 83 N in total or when the maximal load tolerance of the participant had been reached (pain or muscle contraction) (Fig. 2). Consistent with previous work [12], a rest period of approximately 1 min was provided between trials.

Prior to data collection, each session began with a familiarization procedure to determine the maximal tolerable load. Participants first experienced the unloaded roller (~17 N) from S1 to T12. Additional mass was then added in ~11 N increments until a maximum of ~ 83 N or the maximal tolerable load for each participant was reached.

**Fig. 3:**
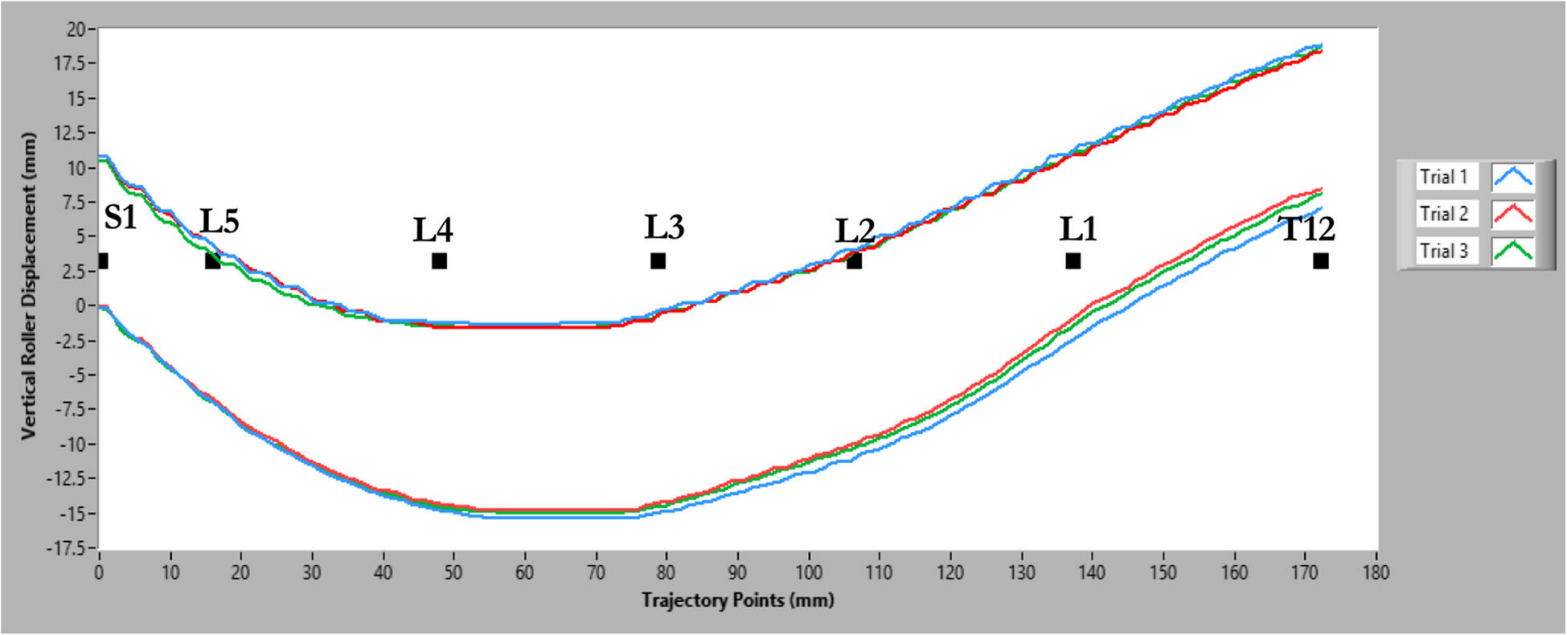
An example of VerteTrack data output as its rollers move over the back and how that data changes with increased applied loading. Three trials are shown for the unloaded condition and three for the maximal tolerable load

Following the familiarization procedure, three trials were conducted per session using the unloaded condition and then three additional trials at the maximal tolerable load condition. Data from these trials were used in the reliability analysis. Figure 3 shows an example of VerteTrack data output as its rollers move over the back and how the data changes with increased applied loading.

In addition, before and during the session, participants were asked to rate any testing-related pain using the NPRS. A reported NPRS of ≥2/10 would stop the loading and the prior mass would be considered as the maximum tolerable load [13].

These same procedures were repeated in the second session including the familiarization procedure and the reliability tests. All tests were conducted by the same examiner who was blinded to the stiffness assessment results of the first session. Between sessions, participants were asked to 1) maintain their usual physical activities and notice if any new activities had been undertaken between sessions or if new symptoms were present, and 2) to not wash the spinous process markings on their body so they could be used in the second session.

### Data analysis of spinal stiffness

The displacement value for each segment was automatically extracted from a custom program written in LabView and then exported to an Excel file. The roller landing and lifting trajectory points (S1 and T12) of all participants were discarded from the automated extracted data. From the remaining continuous displacement data, stiffness was determined at each of the lumbar spinous process locations with the unloaded roller mass defined as the weight of the apparatus (~ 17 N) and the maximum tolerable load considered as the maximum mass that participants could tolerate with no pain and discomfort (~ 61, 72 or 83 N) obtained from the familiarization process. Stiffness at each spinous process location was then calculated as a ratio between the applied force and the resultant displacement [10].

### Statistical analysis

An Intraclass Correlation Coefficient (ICC _3, k_) was calculated to estimate the within-session reliability and the between-session reliability for stiffness values at each lumbar segment separately. ICC with k indicating 1 provided estimates of the relative reliability for a single trial, and at k = 3 provided estimates of the relative reliability for the average of 3 trials. This model of ICC was chosen because only one examiner was involved in this study, representing a fixed factor for rater [14].

Absolute reliability was obtained by calculating the standard error of measurement (SEM) which is defined as an estimation of the variability expected for observed values when the actual value is held constant [15]. The following formula was used:

$$ \mathrm{SEM}=\mathrm{pooled}\ \mathrm{standard}\ \mathrm{deviation}\times \surd \left(1\hbox{-} \mathrm{ICC}\right) $$

**Fig. 4:**
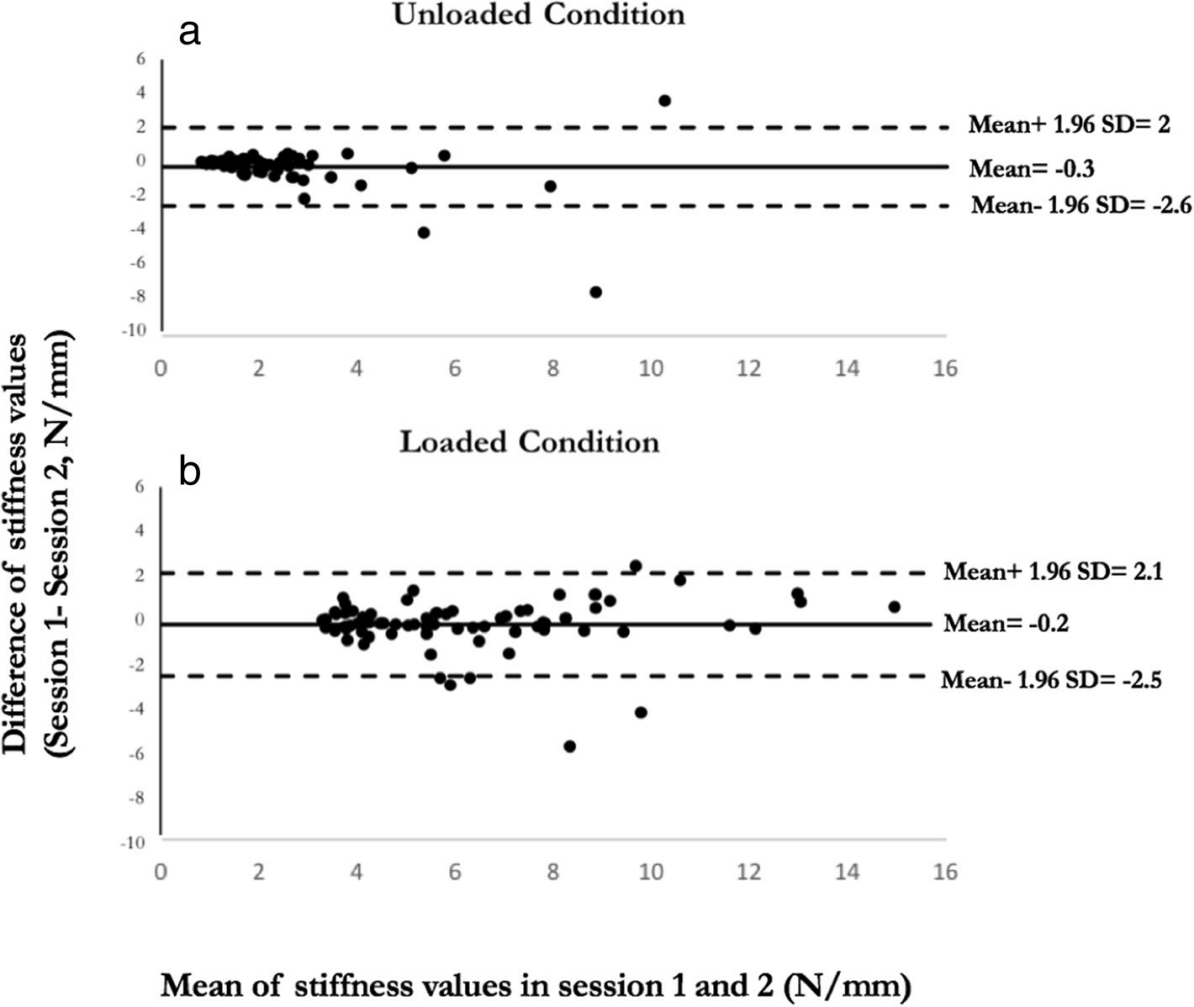
Bland-Altmanplots for between- session agreement in spine stiffness measurements. The central horizontal bias reference lines show the average difference between the measurements between the two testing sessions for the (**a**) unloaded and (**b**) loaded conditions. Outer lines show the limits of agreement (Bias ±1.96* standard deviation)

Bland and Altman graphs were plotted using the difference in spinal stiffness values between session 2 and session 1 (1 minus 2) against the mean of the 2 test sessions to provide a visual presentation of stiffness variability (Fig. 4) [16]. The potential improvement in error when using a single trial or an average of all three trials in determining stiffness was analyzed by comparing the corresponding SEMs.

All statistical analyses were performed using IBM SPSS statistics, version 24 (Armonk, New York, USA), (alpha = 0.05). Intraclass Correlation Coefficient values were qualitatively interpreted using the following criteria: 0.00–0.50 = poor, 0.50–0.75 = moderate, 0.75–0.90 = good, and 0.90–1.00 = excellent [14].

## Results

Seventeen asymptomatic participants, aged 19–43, and homogeneous in terms of age and body mass index were recruited in this study (Table 1). No participant was excluded because of not tolerating the testing procedure. As this study was inclusive of asymptomatic participants only, data from two participants were removed from session 2 due to the development of back pain between the first and second sessions.

**Table 1:** Description of the participants

Characteristic	All participants (*n* = 17)	Male (*n* = 8)	Female (*n* = 9)
Age (years)	29.2 (6)	29.4 (8.4)	29 (3.1)
Height (cm)	171.3 (14.2)	181.1 (13.4)	162.6 (8)
Weight (kg)	68.5 (15.8)	80.9 (13.3)	57.4 (7.1)
Body mass index (kg/m2)	23.0 (2.4)	24.5 (1.7)	21.7 (2.3)
Numeric pain rating scale (/10)- sessions 1	0.35 (0.7)	0.6 (0.9)	0.1 (0.3)
Numeric pain rating scale (/10)- sessions 2	0.24 (0.4)	0.4 (0.5)	0.1 (0.3)

Values are reported as mean (SD)

The within-session reliability (ICC_3,3_) for the single measures was estimated from 0.92 to 1.00 for the unloaded condition and from 0.95 to 1.00 for max tolerable load. In addition, the within-session reliability estimates (ICC_3,1_) for the average of the 3 lumbar spine stiffness measurements ranged from 0.97 to 1.00 for the unloaded condition and from 0.98 to 1.00 for maximal tolerable load (Table 2). The between-session reliability analysis for the first trial of each session (ICC_3,1_) ranged from 0.81 to 0.94 for the unloaded condition and from 0.83 to 0.92 for maximal tolerable load. The between-session reliability estimates of lumbar spine stiffness measurements for the mean of 3 trials (ICC_3,1_) also ranged from 0.75 to 0.96 and 0.82 to 0.93 for unloaded and maximal tolerable load, respectively (Table 2). Overall, the within-session reliability of lumbar spine stiffness measurements was excellent and the between-session reliability was good to excellent after removing two participants who reported having back pain.

**Table 2:** Within-session and between-session reliability of stiffness measurements for lumbar tests

		Minimum Load			Max Tolerable Load		
		Mean (SD) of stiffness values (N/mm)	ICC_3, 1_ (95%CI)/Single measures	ICC_3, 3_ (95%CI)/Average measures	Mean (SD) of stiffness values (N/mm)	ICC_3, 1_ (95%CI)/Single measures	ICC_3, 3_ (95%CI)/Average measures
Within- session Reliability (session 1/ N = 17)	L5	2.6 (1.3)	0.99 (0.97_ 0.99)	1.00 (0.99_ 1.00)	9.0 (3.4)	0.99 (0.97_ 0.99)	1.00 (0.99_ 1.00)
	L4	2.0 (1.5)	0.99 (0.97_ 1.00)	1.00 (0.99_ 1.00)	6.1 (3.0)	1.00 (0.99_ 1.00)	1.00 (0.99_ 1.00)
	L3	2.0 (1.9)	0.92 (0.82_0.97)	0.97 (0.93_ 0.99)	6.2 (5.0)	0.95 (0.89_ 0.98)	0.98 (0.96_ 0.99)
	L2	2.2 (1.7)	1.00 (0.99_ 1.00)	1.00 (0.99_ 1.00)	6.1 (4.4)	1.00 (0.99_ 1.00)	1.00 (0.99_ 1.00)
	L1	2.7 (2.3)	0.98 (0.95_ 0.99)	0.99 (0.98_ 1.00)	7.4 (4.3)	0.99 (0.98_ 1.00)	1.00 (0.99_ 1.00)
Within- session Reliability (session 2/ *N* = 15)	L5	2.3 (0.8)	0.99 (0.97_ 0.99)	1.00 (0.99_ 1.00)	8.1 (2.3)	0.98 (0.96_ 0.99)	1.00 (0.99_ 1.00)
	L4	1.6 (0.6)	0.99 (0.98_ 1.00)	1.00 (0.99_ 1.00)	5.2 (1.6)	0.99 (0.98_ 1.00)	1.00 (0.99_ 1.00)
	L3	1.6 (0.6)	0.99 (0.98_ 1.00)	1.00 (0.99_ 1.00)	5.0 (1.6)	0.99 (0.98_ 1.00)	1.00 (0.99_ 1.00)
	L2	2.4 (2.0)	0.96 (0.90_ 0.98)	0.99 (0.96_ 0.99)	5.9 (3.0)	0.99 (0.98_ 1.00)	1.00 (0.99_ 1.00)
	L1	3.0 (2.4)	0.96 (0.90_ 0.99)	0.99 (0.96_ 1.00)	7.1 (3.4)	0.98 (0.94_ 0.99)	0.99 (0.98_ 1.00)
			ICC_3, 1_ using the first trial	ICC_3, 1_ using the mean of the 3 trials		ICC_3, 1_ using the first trial	ICC_3, 1_ using the mean of the 3 trials
Between- session Reliability (N = 15)	L5	2.2 (0.7)	0.81 (0.54_ 0.93)	0.75 (0.41_ 0.91)	8.1 (2.3)	0.85 (0.60_ 0.95)	0.82 (0.55_ 0.94)
	L4	1.5 (0.5)	0.85 (0.63_ 0.95)	0.84 (0.60_ 0.94)	5.2 (1.6)	0.87 (0.66_ 0.96)	0.93 (0.80_ 0.98)
	L3	1.5 (0.6)	0.88 (0.67–0.96)	0.86 (0.65_ 0.95)	5.0 (1.8)	0.86 (0.62_ 0.95)	0.88 (0.67_ 0.96)
	L2	2.3 (1.9)	0.94 (0.82_ 0.98)	0.96 (0.86_ 0.99)	5.5 (2.6)	0.83 (0.57_ 0.94)	0.86 (0.63_ 0.95)
	L1	2.8(2.6)	0.88 (0.68_ 0.96)	0.75 (0.38_ 0.92)	6.9 (3.5)	0.92 (0.75_ 0.97)	0.89 (0.70_ 0.96)

*Abbreviations*: *SD* standard deviation, *CI* confidence interval, *ICC* intraclass correlation coefficient

The effect of averaging a different number of multiple trials on measurement error (standard error of measurements) shows that averaging three repeated measurements reduced the SEM by a mean of 35.2% over all measurement conditions (Table 3).

**Table 3:** Changes in standard error of measurement (SEM)

		Single Trial		3 Trials		Mean of 3 Trials (% decrease from 1 measure)	
		Min Load	Max Load	Min Load	Max Load	Min Load	Max Load
Within- session SEM (N/mm) _ session 1	L5	0.2	0.4	0.1	0.2	55.0	40.0
	L4	0.2	0.2	0.1	0.1	50.0	55.0
	L3	1.1	1.6	0.7	0.9	39.1	41.3
	L2	0.3	0.4	0.2	0.2	36.7	52.5
	L1	0.9	0.7	0.5	0.4	42.2	45.7
Within- session SEM (N/mm) _ session 2	L5	0.1	0.3	0.1	0.2	30.0	33.3
	L4	0.1	0.2	0.04	0.1	60.0	50.0
	L3	0.1	0.3	0.03	0.2	70.0	50.0
	L2	0.4	1.0	0.2	0.6	40.0	43.0
	L1	0.8	0.4	0.6	0.3	20.0	30.0
Between- session SEM (N/mm)	L5	0.3	1.0	0.3	0.7	0.0	30.0
	L4	0.2	0.6	0.2	0.4	0.0	33.3
	L3	0.2	0.7	0.2	0.5	0.0	28.6
	L2	0.4	0.6	0.3	0.6	25.0	0.0
	L1	1.3	1.2	0.9	0.9	30.8	25.0

*Abbreviations*: *SEM* standard error of measurements

## Discussion

In this study, we evaluated the test-retest reliability of spinal stiffness measurements in asymptomatic individuals using a new device that collects continuous measurements from all lumbar levels and found excellent within- and between-session reliability at the maximal tolerable load. No control group was required for the design of this study.

### Within- and between- session reliability

Our within-session reliability values for stiffness measurement are similar to prior data reported by Wong et al. (ICC, 0,99) [17], and comparable to other studies using single point indentation devices (ICC, 0.96 to 0.98) [18–20]. However, the between-session reliability values at the maximal tolerable load for the averaged measurements (0.90 to 0.94) are lower than Wong et al’s prior study [17] (0.98) but better than those reported from the previous automated techniques (0.85 and 0.88) [20, 21]. The improved between-session reliability of mechanical indenter in Wong et al.’s study might be attributed to his larger sample size. In addition, while Wong et al. used ultrasound to identify the spinous process location, we used an alternative technique by asking each participant not to wash our spinous process markings on their body so they could be used in the next session. We selected this technique as it is not susceptible to ultrasound operator error between sessions – the same markings are used in each participant for each session. Importantly, even if these markings are incorrect in terms of the spinous processes identified, using the same markings are better suited to this reliability study. Therefore, the between-session reliability will not have been affected by the verification of the spinous process location using a traditional manual technique.

Bland and Altman plots show the majority of observations fall on or very near the mean resulting in a high level of agreement between the two measurement sessions. Any difference in stiffness between sessions may be attributed to individual differences between sessions or individual activities of the participants between sessions. Bland and Altman plots also show less reliability at higher stiffness measurements in both unloaded and loaded conditions. Possible explanations for this observation between sessions may include a variety of patient-based factors such as activity level and apprehension level.

### Loaded versus unloaded conditions

The unloaded conditions and the loaded (maximal tolerable load) conditions did not differ significantly in terms of within-session and between-session reliability. This is shown by the ICC confidence intervals presented in Table 2 which overlap for most corresponding estimates for the unloaded and loaded conditions. This suggests that the device provided reliable values regardless of the applied load. However, for the majority of the comparisons between the corresponding unloaded and loaded ICC point estimates, when there is a difference, the point estimate of the loaded condition is better. Clinically, the unloaded condition will likely be more tolerable in patients with LBP and our results confirm that the unloaded condition can provide reliable data.

### Changes in measurement error by multiple trial

Our study found that using an average of the three trials to create within-session stiffness values showed a reduction in SEMs as compared with a single trial. This is consistent with previous studies [17] that showed using an average of three measurements improved the measurement error. Therefore, we suggest taking the results from an average of 3 trials if possible to calculate the stiffness of a spinal region using VerteTrack.

### Limitations and future research

The study protocol, which was designed for a research study on reliability, took 30–45 min including the familiarization procedure. Using single trials only, the total time to complete testing is ~ 12 min [22].

While participants returned at similar times on separate sessions, it is currently unclear whether better control of inter-session time intervals and/or activities would improve between-session reliability results; it is impossible to know if a change in reliability in the second session is the result of differences in the participant over time, variability in the measurement process, or both. This is a drawback of reliability testing over multiple days. Furthermore, the measures obtained by a loading device such as this will always be influenced by the viscoelastic properties of the target tissues in their current state. As such, the reliability of this device is dependent on providing adequate recovery time between trials.

While we expect that the reliability of the device may change when used to evaluate spinal pathology, this device may be contraindicated in specific pathologies as well (e.g. fracture, metastatic disease). Further studies are needed to define relative and absolute contraindications for VerteTrack use. It is important to note that the reliability of the VerteTrack is likely decreased by patient-based factors such as voluntary/involuntary muscle contraction, changes in patient position during testing and inconsistent patient breathing procedures. Future identification of these factors and the magnitude of their impact is warranted.

## Conclusions

This study evaluated the reliability of a device capable of measuring spinal stiffness continuously over an entire spinal region in asymptomatic human participants. The new technique was shown to produce reliable measurements in quantifying load-displacement values for within-session and between-session assessments. The resulting data may have greater clinical utility than single site measures in that spinal stiffness can be obtained not only at one level, but over the entire spinal region of interest.

## Abbreviations

ICC: Intraclass correlation coefficients
LBP: Low back pain
NPRS: Numerical pain rating scale
SEM: Standard error of measurement

## References

[1] Latimer J, Lee M, Adams R, Moran CM (1996). An investigation of the relationship between low Back pain and lumbar Posteroanterior stiffness. J Manip Physiol Ther.

[2] Snodgrass SJ, Haskins R, Rivett DA. A structured review of spinal stiffness as a kinesiological outcome of manipulation: its measurement and utility in diagnosis, prognosis and treatment decision-making. J Electromyogr Kinesiol. 2012;22(5):708–723. [cited 2016 Jan 15] 10.1016/j.jelekin.2012.04.015 22683056

[3] Wong AYL, Kawchuk GN. The clinical value of assessing lumbar Posteroanterior segmental stiffness: a narrative review of manual and instrumented methods. PM R; 2016. 10.1016/j.pmrj.2016.12.001 27993736

[4] Childs JD, Fritz JM, Flynn TW, Irrgang JJ, Johnson KK, Majkowski GR, et al. A clinical prediction rule to identify patients with low back pain most likely to benefit from spinal manipulation: a validation study. Ann Intern Med 2004;141(12):920–928. 10.7326/0003-4819-141-12-200412210-00008 15611489

[5] Stanton TR, Kawchuk GN. Reliability of assisted indentation in measuring lumbar spinal stiffness. Man Ther. 2009;14(2):197–205. [cited 2015 Nov 22] 10.1016/j.math.2008.01.011 18375172

[6] Latimer J, Lee M, Adams RD (1998). The effects of high and low loading forces on measured values of lumbar stiffness. J Manip Physiol Ther.

[7] Caling B, Lee M (2001). Effect of direction of applied mobilization force on the posteroanterior response in the lumbar spine. J Manip Physiol Ther.

[8] Adams R. A psychophysical evaluation of manual stiffness discrimination. Aust J Physiother. 1995;41(3):161–167. Available from: 10.1016/S0004-9514(14)60426-8. 10.1016/S0004-9514(14)60426-8 25026039

[9] Wong AYL, Parent EC, Dhillon SS, Prasad N, Kawchuk GN. Do participants with low Back pain who respond to spinal manipulative therapy differ biomechanically from nonresponders, untreated controls or asymptomatic controls? Spine (Phila Pa 1976). 2015;40(17):1329–1337. 10.1097/BRS.0000000000000981 26020851

[10] Fritz JM, Koppenhaver SL, Kawchuk GN, Teyhen DS, Hebert JJ, Childs JD. Preliminary investigation of the mechanisms underlying the effects of manipulation: exploration of a multivariate model including spinal stiffness, multifidus recruitment, and clinical findings. Spine (Phila Pa 1976) 2011;36(21):1772–1781. 10.1097/BRS.0b013e318216337d PMC3150636 21358568

[11] Walter SD, Eliasziw M, Donner A (1998). Sample size and optimal designs for reliability studies. Stat Med.

[12] Brown BT, Blacke A, Carroll V, Graham PL, Kawchuk G, Downie A (2017). The comfort and safety of a novel rolling mechanical indentation device for the measurement of lumbar trunk stiffness in young adults. Chiropr Man Therap.

[13] Childs JD, Piva SR, Fritz JM (2005). Responsiveness of the numeric pain rating scale in patients with low back pain. Spine (Phila Pa 1976).

[14] Koo TK, Li MY. A guideline of selecting and reporting Intraclass correlation coefficients for reliability research. J Chiropr Med; 2016;15(2):155–163. Available from: 10.1016/j.jcm.2016.02.012 10.1016/j.jcm.2016.02.012 PMC4913118 27330520

[15] Dudek FJ (1979). The continuing misinterpretation of the standard error of measurement. Psychol Bull.

[16] Bland JM, Altman DG (1999). Measuring agreement in method comparison studies. PubMed Commons.

[17] Wong AYL, Kawchuk G, Parent E, Prasad N. Within- and between-day reliability of spinal stiffness measurements obtained using a computer controlled mechanical indenter in individuals with and without low back pain. Man Ther. 2013;18(5):395–402. [cited 2015 Oct 8] 10.1016/j.math.2013.02.003 23465962

[18] Latimer J, Goodsel MM, Lee M, Maher CG, Wilkinson BN, Moran CC. Evaluation of a new device for measuring responses to posteroanterior forces in a patient population, part 1: reliability testing. Phys Ther 1996;76(2):158–165. 10.1093/ptj/76.2.158 8592719

[19] Edmondston SJ, Allison GT, Gregg CD, Purden SM, Svansson GR, Watson A. E. Effect of position on the posteroanterior stiffness of the lumbar spine. Man Ther 1998. 3 p. 21–26. Available from: 10.1054/math.1998.0312 11487297

[20] Shirley D, Ellis E, Lee M (2002). The response of posteroanterior lumbar stiffness to repeated loading. Man Ther.

[21] Lee M, Svensson NL. Measurement of stiffness during simulated spinal physiotherapy. Clin Phys Physiol Meas 1990;11(3):201–207. 10.1088/0143-0815/11/3/002 2245584

[22] Young A. Validating assessment of spinal stiffness: bench-top performance of the VerteTrack system: Masters Res thesis, Macquarie Univ North Ryde NSW; 2019.

